# Contribution of Congenital Heart Disorders Associated With Copy Number Variants in Mediating Risk for Brain Developmental Disorders: Evidence From 20-Year Retrospective Cohort Study

**DOI:** 10.3389/fcvm.2021.655463

**Published:** 2021-07-15

**Authors:** Luke Dowden, David Tucker, Sian Morgan, Orhan Uzun, Yasir Ahmed Syed

**Affiliations:** ^1^Neuroscience and Mental Health Research Institute, Cardiff University, Cardiff, United Kingdom; ^2^School of Bioscience, Cardiff University, Cardiff, United Kingdom; ^3^Knowledge Directorate, Public Health Wales, Swansea, United Kingdom; ^4^Department of Medical Genetics, University Hospital of Wales, Cardiff, United Kingdom; ^5^Department of Fetal Cardiology, University Hospital of Wales, Cardiff, United Kingdom

**Keywords:** copy number variants, congenital heart disorder, neurodevelopmental disorders, 22q11 deletion syndrome, retrospective study

## Abstract

Rare pathogenic copy number variants (CNVs) are genetic rearrangements that have been associated with an increased risk for congenital heart disorders (CHDs). However, the association of CNVs with atypical brain development, leading to neurodevelopmental disorders (NDDs), in the presence of CHDs remains unclear. We attempted to explore this association by establishing the prevalence and burden of CNVs associated with CHD in a Welsh population and by studying the effect of rare CNVs associated with CHDs in mediating the risk of NDDs. Toward this goal, we analyzed data from the Congenital Anomaly Register for Wales (CARIS), referred from hospitals in Wales between 1998 and 2018, which included 1,113 subjects in total. Of these, 785 subjects were included in the study following application of the exclusion criteria, and a total of 28 rare CNVs associated with CHD were analyzed. The findings from this cohort study identified 22q11.2 deletion as the most prominent CNV across the cohort. Our data demonstrates that the survival rate of the cohort after 3 years was 99.9%, and mortality fell significantly between 1 and 2 years and between 2 and 3 years [*F*_(1,27)_ = 10, *p* = 0.0027; *F*_(1,27)_ = 5.8, *p* = 0.0222]. Importantly, the data set revealed a positive correlation between the incidence of congenital heart disease and the incidence of neurodevelopmental abnormalities in patients with CNVs across the whole cohort [95% CI (0.4062, 0.8449), *p* < 0.0001, *r* = 0.6829]. Additionally, we identified significant CNVs that result in the co-morbidity of CHD and NDD and show that septal defects and global developmental delay are major congenital defects. Further research should identify a common molecular mechanism leading to the phenotypic comorbidity of CHDs and NDDs, arising from a common CNV, which can have an implication for improving risk classification and for fetal neuroprotection strategies in the affected children and in precision medicine.

## Introduction

Congenital heart disorders (CHDs) represent the most common group of medically significant birth anomalies (1), yet, in ~80% of cases, the underlying cause remains unknown (2). In ~20% of CHD patients, chromosomal anomalies or gene defects appear to be the causal factor (3). Copy number variations (CNVs), resulting from a non-allelic homologous recombination, result in deletions or duplications of chromosomal loci and are considered an important genetic risk factor for CHD (4, 5). Chromosomal regions 1q21.1, 15q11.2, 15q13.3, 16p11.2, 16p13.11, and 22q11.2 harbor rare but recurrent and highly penetrant CNVs that have been uncovered as important risk factors for CHD (5–9). A number of genome-wide association studies across different populations have also revealed these CNVs to be associated with neurodevelopmental abnormalities (10, 11). However, it is far from clear as to what extent patients with CNV-associated congenital heart disorders are at risk of developing neurodevelopmental disorders. Previous evidence from epidemiological studies points toward neurodevelopmental disorder (NDD) incidence in ~10–20% of all CHD patients (12, 13). Furthermore, with increased survival outcome (>90%) in patients with congenital heart disorders (14), more attention is being focused on improving their quality of life, as these individuals are suspected to be at a higher risk for developing cognitive and learning disabilities (15–17). Hence, it is important to establish the association between these two developmental disorders in patients where associated copy number variants are a common factor.

In this study, we aim to address this fundamental gap in knowledge by studying an all-Wales clinical cohort. Data, constituting 1,113 patients, was collected over a period of 20 years. In particular, we were interested in the landscape of congenital heart disorders stemming from copy number variants. Furthermore, we aimed to determine the association between the incidence of a congenital heart disease and the incidence of a neurodevelopmental disorder in patients carrying CNVs, investigate the burden of these CNVs in patients, and identify CNVs which result in the highest co-morbidity of these two clinically important development disorders. We further specifically hypothesized that there would be an excess of NDDs in CNV-associated CHD patients, as compared to CHD patients in general, due to the contribution of CNV loci in developmental biological processes.

## Methods

### Data Source and Study Design

This study was a retrospective cohort study using data collected by the Congenital Anomaly Register and Information Service for Wales (CARIS). The registry began data collection in 1998 on an all-Wales basis. The registry uses multiple sources of ascertainment and routinely receives notifications from over 25 independent sources. Of particular importance to this study were data received from the All Wales Genetics Laboratory, hosted at the University of Wales Health Board (based in Cardiff), data from the pediatric cardiology databases (Cardiff & Liverpool), the inpatient database (PEDW), and pediatric clinic letters, accessed either through hospital clinical portal systems or through a direct review of patient case notes. The data was processed using a standard methodology, as set out in EUROCAT guide 1.4 [https://eu-rd-platform.jrc.ec.europa.eu/eurocat/data-collection/guidelines-for-data-registration_en (accessed June 2021)], to ensure uniformity of case definitions, data set variables, and clinical coding practice with similar registers across Europe. Congenital anomalies were coded using ICD10, and for rare diseases (which CNVs are), Orphanet codes [https://www.orpha.net/consor/cgi-bin/index.php (accessed June 2021)] were also used to allow greater discrimination than is possible with ICD10. NDD data was collected as co-morbidities along with the congenital anomaly data and from the same sources as outlined above. This is often of a general descriptive character, e.g., “developmental delay,” rather than a precise diagnosis. Therefore, ascertainment of NDDs is unlikely to be complete.

The majority of referrals occurred between 2010 and 2015, with a general increase in referrals between 1998 and 2010 and then a general decrease in referrals between 2015 and 2018 (Supplementary Figure 1).

### Identification of Chromosomal Abnormalities

The chromosomal abnormalities described in this study were reported by the All Wales Genetics Laboratory and submitted to the CARIS database. Owing to the 20-year period, the abnormalities were detected using differing technologies, including the use of karyotyping, fluorescence *in situ* hybridization, and microarray (using an oligonucleotide platform).

### Processing of Data

**Figure 1:**
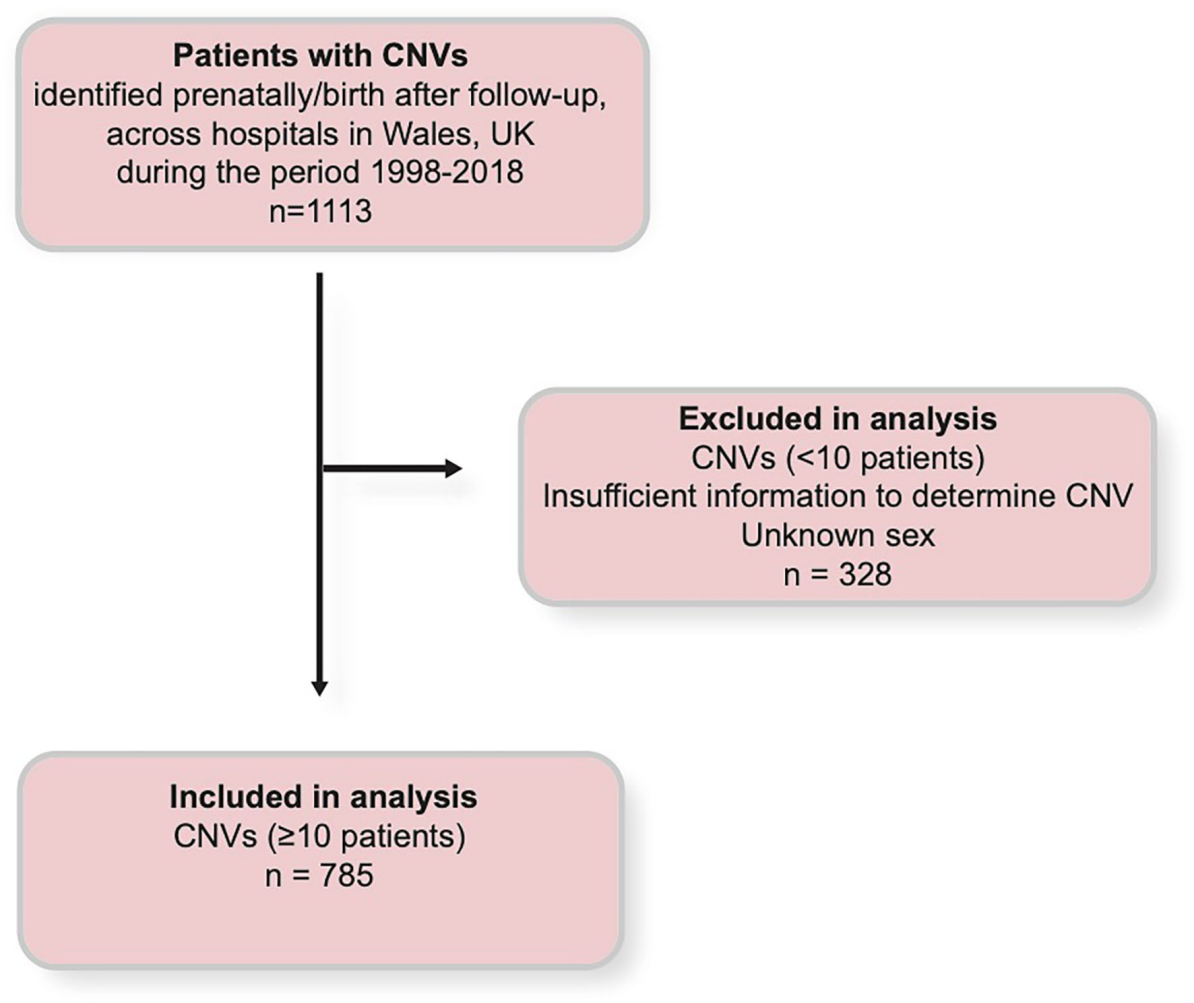
Chart illustration of the selection criteria by which data was obtained and used, and by which data was excluded during analysis. CNV, copy number variant.

Information in this data set included CNV type (including multiple CNVs), CNV length, birth date, date of death (where applicable), sex, and the details of congenital malformations (Supplementary Table 4). 5p deletions in this data set are of the range 5p13-15.2. Additionally, “2q deletion” represents a variety of deletions of the range 2q11.1–2q37.3, and “Xq duplication” represents a variety of duplications on the long arm of the X chromosome, most of which at an unspecified locus. Exclusion criteria were employed where diagnosis of CNV type (such as chromosome number and the nature of the change at the CNV locus) could not be determined and for patients terminated or lost before their sex was ascertained (the remaining patients lost prenatally were being recorded as having been lost within 1 year following the end of pregnancy along with post-natal deaths up to 1 year following the end of pregnancy). CNV types which were represented by fewer than 10 patients were also excluded from analysis due to lack of power for statistical analysis (Figure 1).

Many of the patients referred had multiple CNVs. In those patients who harbored multiple CNVs, the locus of the “primary” CNV was determined. This was done on the following criteria: (a) a notable or important CNV as identified in similar studies to have shown to increase the risk for CHDs and (b) in the absence of a notable CNV, the largest CNV (as CNV length has been shown to increase the risk for NDD and CHD) (5). As such, noteworthy pathogenic CNVs were labeled as the primary CNV over minor CNVs (in spite of length) where possible. Furthermore, in the case that multiple pathogenic CNVs were identified in a patient, they were both recorded as primary CNVs. CHDs were categorized into five groups; septum, great vessel(s), left/right patterning, valve(s), and other, as outlined by Costain et al. (4). NDDs were categorized into six groups: intellectual disability, communication disorders, autism spectrum disorders, motor disorders, global developmental delays, and other. These groups were adapted from the DSM Manual−5th Edition. Diagnosis of neurodevelopmental characteristics was performed by experienced general and community pediatricians using conventional assessment tools. Where necessary (and specifically in the cases of the autistic spectrum and communication disorders), expert panel decisions, involving clinicians and appropriate therapists, were employed for this purpose.

### Functional Analysis of Genes

To expand on our analysis, we carried out a PANTHER search to identify the functional classifications of genes at each of the four CNV sites associated with the highest coincidence of CHD and NDD [http://pantherdb.org/ (accessed June 2021); (18)]. The genes at each locus were gathered from sources in the literature, and the genes used in this search were limited to those known to be expressed in the heart or brain or in both (Supplementary Table 2). Identification of such genes was done via the “Evo-devo mammalian organs” online application, developed by the Kaessmann lab [https://apps.kaessmannlab.org/evodevoapp/ (accessed June 2021); (19)].

### Statistical Analysis

Statistical analysis of the data was done in GraphPad Prism, version 8.4 (GraphPad Software, San Diego, California, USA). Two-sided *p* < 0.05 were considered significant. A one-way ANOVA was used to investigate the difference between mean CNV lengths. A two-way ANOVA test was conducted to investigate patient mortality between 1 year (including fetal losses, still births, and terminations, with the exception of those lost before their sex could be determined) and 2 years following the end of pregnancy. Patient mortality was transformed with the addition of a random Gaussian value, with a standard deviation of 0.1. Two-way ANOVA of this data was conducted with a Geisser–Greenhouse correction, as sphericity was not assumed. A second two-way ANOVA was conducted with the same transformed data to examine the reduction in mortality between 2 and 3 years following the end of pregnancy. A Spearman's correlation test was used to investigate the correlation between the incidence of CHD and the coincidence of CHD and NDD. For this analysis, each CNV recorded was treated as a separate observation, and CHD/NDD frequencies were plotted as continuous values (Supplementary Figure 2). A two-way ANOVA test to examine the difference in the number of patients with a CHD only and those with both CHD and NDD was conducted. Values for the incidence of CHD only and values for the coincidence of both CHD and NDD were transformed with the addition of a random Gaussian value, with a standard deviation of 0.1. A two-way ANOVA was run to investigate the differences in CHD category prevalence among patients by CNV. Data for the incidence of each CHD category was transformed with the addition of a random Gaussian variable, with a standard deviation of 0.15. A similar analysis of the incidence of NDD categories was also conducted, in which the data was transformed with the addition of a random Gaussian variable with a standard deviation of 1.05. Two-way ANOVAs were used to investigate the distribution of gene activity in biological processes, both as an absolute value and as a proportion of the sum of gene activity in all biological processes at each CNV locus.

## Results

### Burden and Characteristic Features of CNVs in a Cohort

**Figure 2:**
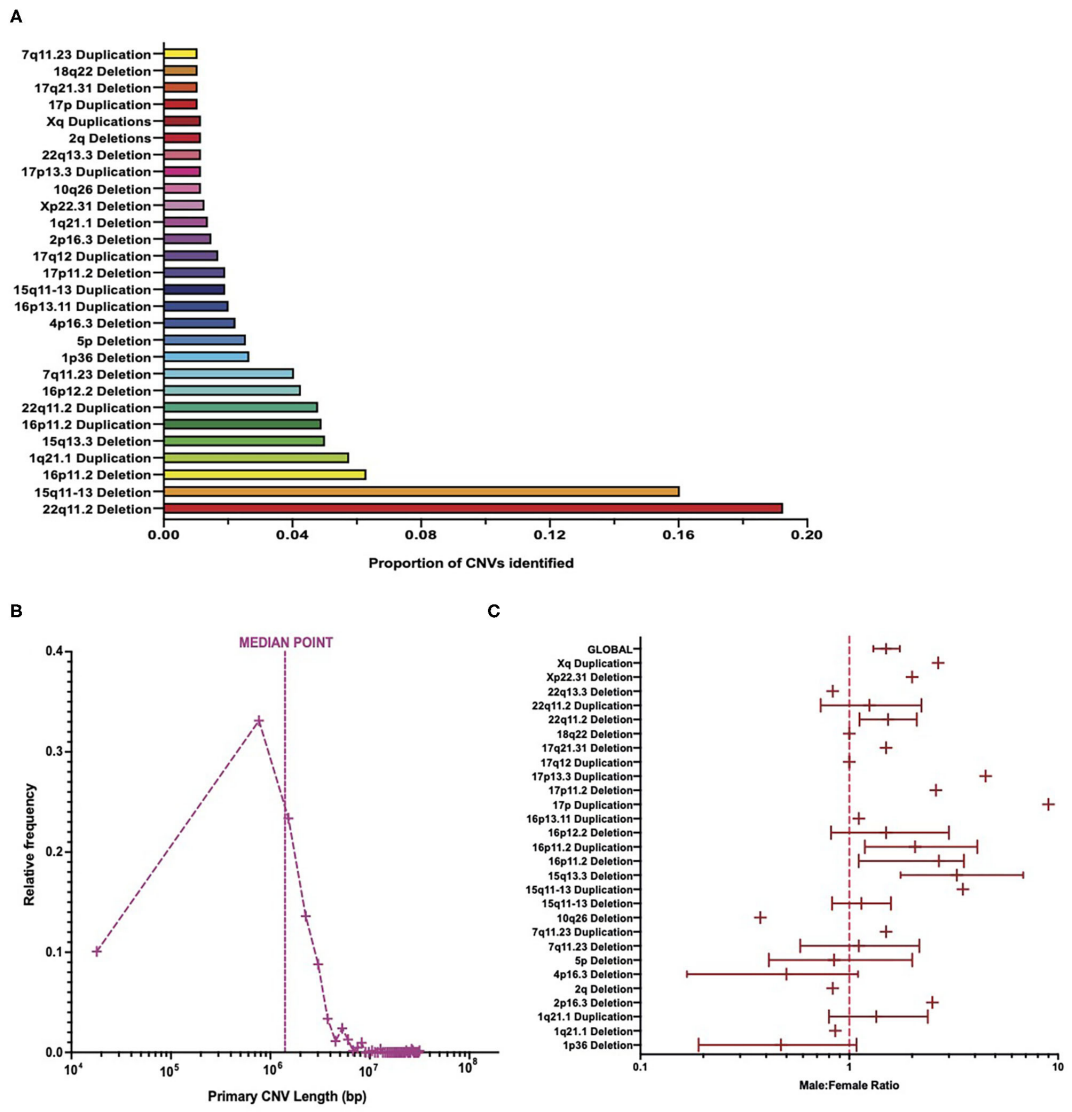
Burden of copy number variants across cohort. **(A)** The number of patients with each CNV type in comparison to the total number of CNVs. **(B)** Frequency distribution of primary CNV identified in patients equating to the total number of primary CNV lengths identified, on a logarithmic axis, with a line signifying the median point. **(C)** Meta-analysis of cohort sex ratio on a logarithmic axis, broken down by each CNV type, with 95% confidence intervals calculated for CNVs ≥20 patients (and global value) via bootstrapping with *N* = 1,000 samples. CNV, copy number variant.

Distribution analysis showed that deletions at the 22q11.2 locus were the most frequently identified CNVs among patient data, representing almost 20% of the total CNVs identified (Figure 2A). Deletions in the 15q11-13 range were the second most frequent CNVs, accounting for about 16% of all CNVs identified (Figure 2A). These two CNVs together account for over a third of the CNVs identified, with subsequent CNVs accounting each for no more than 7% of the total number of CNVs identified (Figure 2A).

The length of primary CNVs identified in the data set varied from 18 kbp to 31 Mbp, with a median point of ~1.4 Mbp (Figure 2B). When placed into groups of 750 kbp (i.e., bin length of 750,000), the highest frequency of CNVs in a single category fell below the median point at ~768 kbp, with ~33% of CNVs represented in this category (Figure 2B). However, due to the large range between the smallest and largest CNV lengths, and the varying frequency of CNV lengths among patients, a large proportion of patients had CNV lengths much larger than the median point. This is an important consideration in light of evidence that suggests a positive correlation between CNV length and severity of disease phenotypes (5, 20).

In total, more male patients were present in this data set than females, with a ratio of ~1.4 male patients for every female patient (Figure 2C). This, however, varied quite considerably between CNV types, with some CNVs being represented by more female patients than male patients, some CNVs having an equal number of male and female patients, and CNVs with a higher number of male patients than female patients varying in the extent of this difference (Figure 2C). That being said, 22q11.2 deletions (the most frequent CNV type identified) had a male/female ratio of ~1.5 (Figure 2C) and thus suggest that a slightly higher number of male patients than female patients is a trend borne out of the data. A two-way ANOVA revealed that there were significantly more males than females [*F*_(1, 27_ = 6.5, *p* = 0.0137] but suggested that this differed between CNVs in a non-significant way [*F*_(27,27)_ = 0, *p* > 0.9999]. This result is interesting, given that previous studies suggest that more females in the general population carry large CNVs than males (21).

The 95% confidence intervals, generated from bootstrapping with *N* = 1,000 samples, appear to suggest differing variability in the sex ratio of patients in this cohort (Figure 2C and Supplementary Table 1), with the variability of sex ratio for some CNVs appearing quite high. The calculation of these confidence intervals is influenced by the sample size, and hence they were not calculated for CNVs with <20 patients. Differing sample sizes may account for the varying range of confidence intervals between CNVs and for the apparently high uncertainty of some CNVs. Supplementary Figure 3 shows the same data on a linear scale for easier comparison of confidence intervals between CNVs and suggests that, for those CNVs with the highest variation in sex ratio, many of them still fall at a male-to-female ratio >1. Additionally, the 95% confidence intervals for the cohort globally are relatively narrow. Therefore, while the variability in sex ratio differs between CNV and appear relatively high for some CNVs, the trend still indicates a higher number of males than females in this cohort.

### CNV Burden Has Little Impact on the Survival Rates of Patients After 2 Years of Age

**Figure 3:**
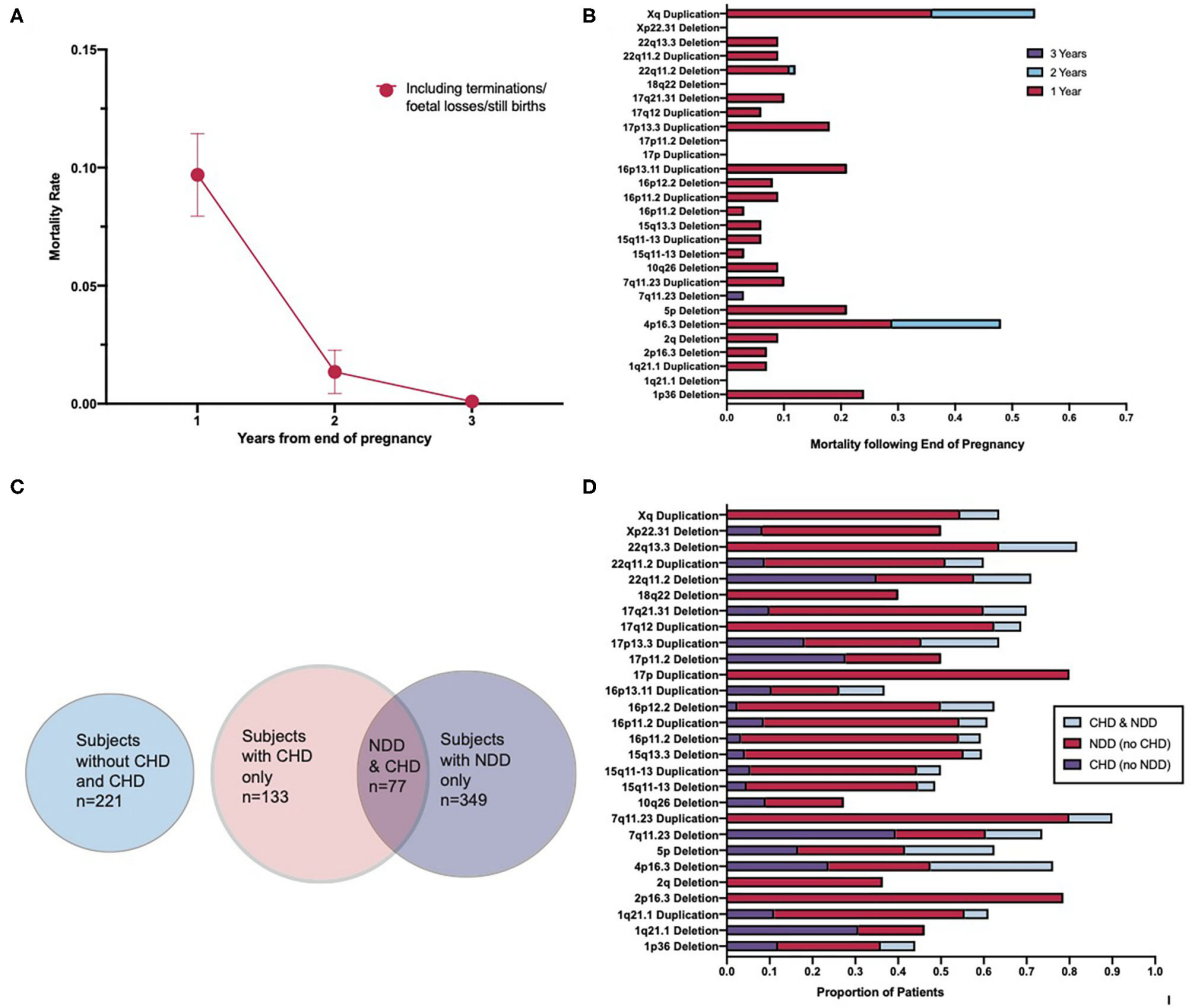
Association of congenital heart disorders and neurodevelopmental disorders resulting from the pleiotropic effect of copy number variants. **(A)** Mortality rate of patients across a 3-year period following end-of-pregnancy. **(B)** Cumulative mortality over a 3-year period by CNV type. **(C)** Venn diagram illustrating the overlap in the number of CHDs and NDDs across the cohort, including a separate indicator of those patients with neither a CHD nor a NDD. **(D)** Percentage distribution of patients with CHDs, NDDs, and with both CHDs and NDDs across each CNV type. Spearman's correlation test for global correlation between presence of CHD and presence of both CHD and NDD revealed a statistically significant positive correlation (*r* = 0.6956, *p* < 0.0001). A two-way ANOVA test revealed the CNV type as a statistically non-significant factor in the correlation between the presence of CHD and both CHD and NDD [*F*_(27,27)_ = 1, *p* = 0.0686]. CNV, copy number variant; CHD, congenital heart disorder; NDD, neurodevelopmental disorder.

Patients that are born appear to be most susceptible to premature mortality during the first year of life (Figures 3A,B). Further to this, the highest mortality rate occurred in patients prenatally and within the first year following end of pregnancy (Figures 3A,B). Mortality falls between 1 and 2 years following birth, and by 3 years following birth, the mortality rate reduces to almost 0 (Figure 3A). A two-way ANOVA revealed that the decrease in mortality between 1 and 2 years following end of pregnancy was statistically significant [*F*_(1,27)_ = 10, *p* = 0.0027] and that the variation in mortality rate between CNV loci was not significant [*F*_(1,27)_ = 0, *p* = 0.5456]. This suggests that the mortality rate between CNVs did not differ enough to imply a role for CNV loci in differing mortality rates and that it is likely that these differences occurred as a consequence of statistical chance. The results of a second two-way ANOVA indicate that the difference in mortality between 2 and 3 years following end of pregnancy was also statistically significant [*F*_(1,27)_ = 5.8, *p* = 0.0222] and that the differences in mortality rate between CNV loci were again non-significant [*F*_(1,27)_ = 1, *p* = 0.3004]. It appears, therefore, that routine surgical intervention intended to correct congenital malformations is effective in reducing the mortality of CNV-carrying patients.

### Copy Number Variants Contribute to Both Congenital Heart Disorders and Neurodevelopmental Disorders

To further explore the relationship between CHDs and NDDs in CNV patients, the coincidence of CHDs and NDDs, across the cohort and by CNV, was examined. Totally, over one-third of those patients in the data set that had a congenital heart disease also had a neurodevelopmental disorder (Figure 3C). While this varies between CNV types (Figure 3D), a Spearman's correlation test revealed a strong, significant positive correlation between the presence of a congenital heart disease and the presence of both a congenital heart disease and a neurodevelopmental disorder globally [95% CI (0.4062, 0.8449), *p* < 0.0001, *r* = 0.6829]. This suggests that CNV patients with a congenital heart disease are a risk group for developing abnormalities associated with aberrant brain development. Previous studies have pointed toward 10–20% of all CHD patients having an associated NDD (12, 13), while our result demonstrates that about 33% of CHD in our cohort were associated with NDD, suggesting an excess of CHD–NDD co-occurrence in CNV patients as compared to the general population. A two-way ANOVA was conducted to examine if the coincidence of CHD and NDD differed significantly between different CNVs and found that the variation between CNVs was non-significant [*F*_(27,27)_ = 1, *p* = 0.0686]. These results suggest that CHD and NDD incidence is correlated in CNV patients, the presence of a CNV in CHD patients increases their risk of developing an NDD as compared to the general CHD patient population, and this is not tied to the effect of any specific CNV in particular. Given how close, however, this result is to being considered statistically significant, future research with other cohorts may reveal a statistically significant relationship between some CNVs and CHD–NDD co-occurrence as compared to other CNVs.

It should be noted that during examination of NDD patients for enrichment of CHD, no correlation was found. This reaffirms the findings of this study and others in that there is an excess of NDDs in CHD patients, but there appears not to be an excess of CHDs in NDD patients. Thus, the excess of NDDs in patients is correlated specifically with the presence of CHDs.

**Figure 4:**
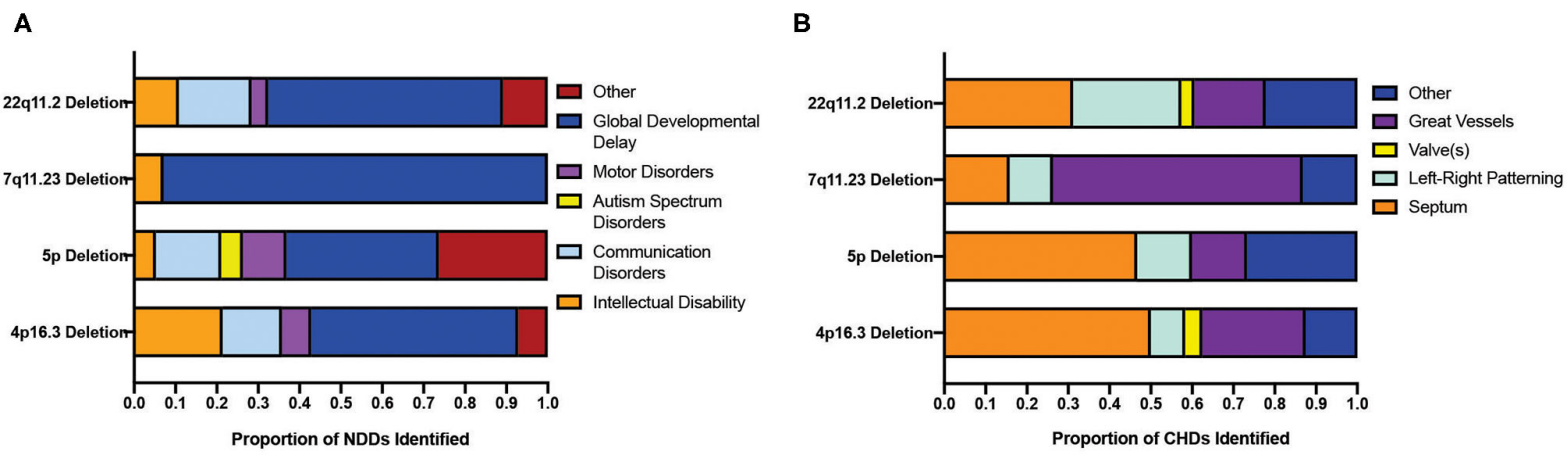
Distribution of sub-clinical phenotypes of neurodevelopmental and congenital disorders across CNVs displaying a significant co-morbidity. **(A)** Distribution of the clinical phenotypes of NDDs across four CNVs showing the highest co-morbidity of NDD and CHDs. **(B)** Distribution of the clinical phenotypes of CHDs in the top four CNV populations showing the highest co-morbidity of NDD and CHD. CNV, copy number variant; CHD, congenital heart disorder; NDD, neurodevelopmental disorder.

Four CNVs (22q11.2 deletion, 7q11.23 deletion, 5p deletion, and 4p16.3 deletion) associated with the highest co-morbidity of CHDs and NDDs were further investigated, and thus the CHDs and NDDs were categorized accordingly (as outlined in the “METHODS”). As such, it appears that the proportion of CHD categories identified varies between CNVs (Figure 4B) but that the NDDs identified are largely dominated by those labeled “global developmental delay,” which includes motor, communication, and intellectual disorders (Figure 4A). This observation may reflect how genes located at different CNV loci vary in their roles in heart development, and thus the disruption of these genes produces disease phenotypes largely inconsistent in nature, and that NDDs constitute a disparate group of conditions that largely overlap in phenotype (limiting the usefulness of the DSM-5 classification) (22).

### Functional Analysis of Significant CNVs

**Figure 5:**
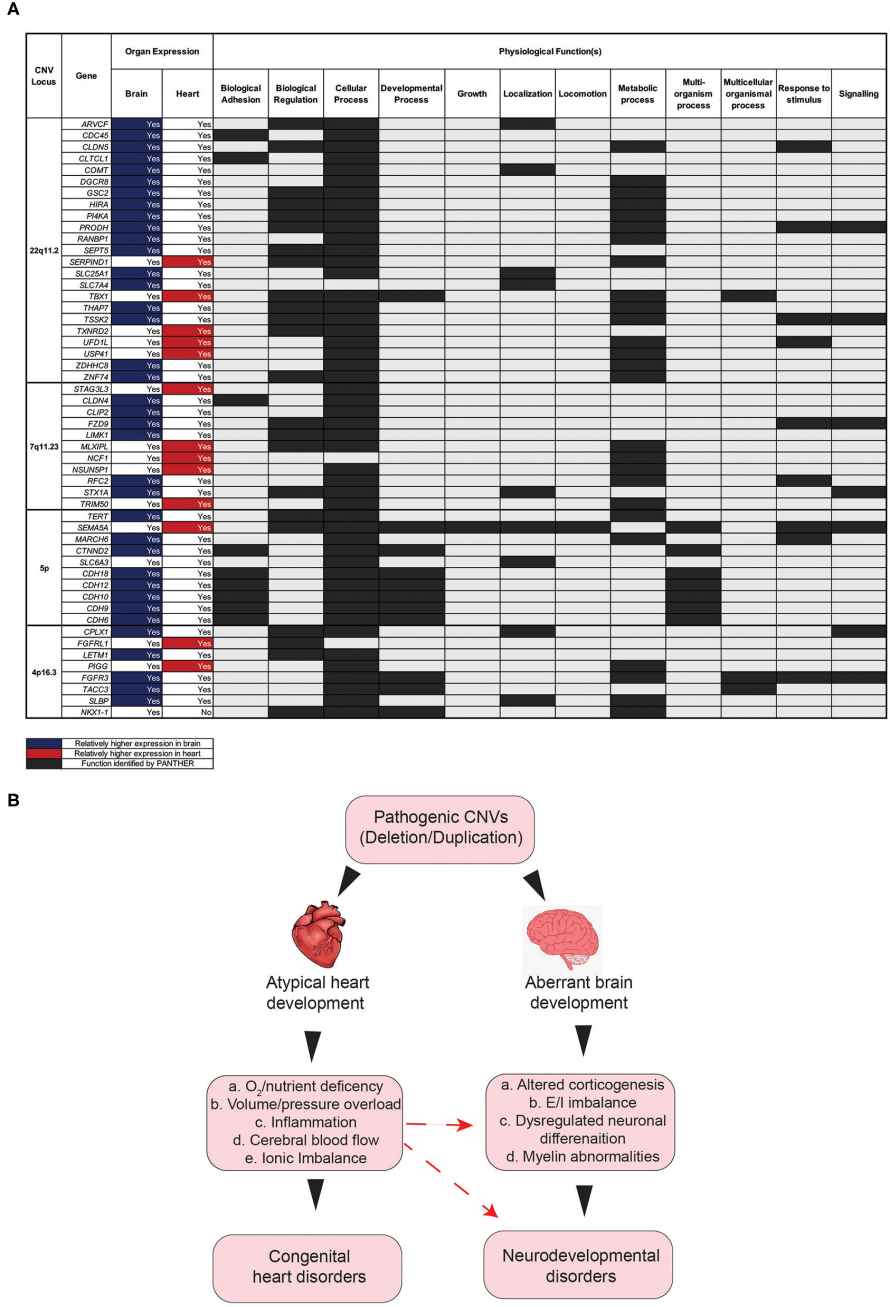
Impact of gene disruption on the onset of aberrant brain and heart development. **(A)** Physiological functions of genes associated in the comorbidity of congenital and neurodevelopmental disorders of four CNVs with high CHD/NDD coincidence. **(B)** Schematic diagram showing the hypothesis as to how the CHD presence in CNV patients can contribute to NDD phenotypes secondarily. CNV, copy number variant; CHD, congenital heart disease; NDD, neurodevelopmental disorder.

To further explore pleiotropic effects at the gene level for each of the top four CNVs, a search was performed with the PANTHER Classification System, intended to identify the functional classification of genes at each of the four CNV loci. Almost all genes inputted from each of the four CNVs were expressed in at least one of the two organs. Of note, however, is that most of these genes were expressed in both organs (Figure 5A). The PANTHER search identified many of the genes inputted as being involved in a variety of biological processes, such as localization, signaling, and regulation (Figure 5A). The definitions of these processes can be found in the Supplementary Table 3.

This search revealed that most genes for the four significant CNVs are involved in a “cellular Process” (Figure 5A). Other notable functions include “metabolic process,” “biological regulation,” “response to stimulus,” and “signaling” (Figure 5A). The results of this search suggest that the genes affected by copy number variation at each of these four loci play important roles in a variety of biological processes that associated with physiology both in the brain and the heart (Figure 5A). It follows, therefore, that the deletion/duplication of genetic locus involved in these functions may critically disrupt pathways involved in both heart and brain development (Figure 5B), potentially by a complex molecular mechanism (23–26). Additionally, disruption to heart development may have secondary consequences for brain development. Several factors, occurring as a consequence of aberrant heart development, may, in turn, exacerbate the onset of neurodevelopmental abnormalities (Figure 5B).

Furthermore, disruption to heart development has been linked to hindered brain development and, in turn, NDD symptoms (27). The heart and the brain develop within the same time window prenatally, and thus reduced blood flow (because of a congenital heart disease), among other consequences of congenital heart malformations, could limit brain development. Therefore, it is likely that the disruption of genes, as a consequence of CNV mutations, contributes to NDD onset, both directly through changes in neuron activity and indirectly through reduced blood flow to the brain during fetal development [Figure 5B; (28)].

## Discussion

### Principal Findings

Our study, for the first time, identifies patients with a CNV and CHD as being a statistically significant risk group for the development of a neurodevelopmental disorder. Although previous studies have independently linked both CHD and NDD to CNVs (4, 11), emerging studies suggest that congenital heart diseases confer a greater risk to neurodevelopmental disorders in non-CNV patients (12, 29, 30). Here we demonstrate a significant relationship between CHDs and NDDs in CNV patients and have identified the types of CNV that induce a pleiotropic effect. Furthermore, we have identified several CNVs with a particularly high coincidence of CHDs and NDDs. With over one-third of the total CNV patients with a CHD also suffering from an NDD, as compared to 10–20% of CHD patients in general (12, 13), these results suggest a role for CNVs in increasing the likelihood of NDD onset in CHD patients. Clinically, this reveals a need for CHD patients, particularly when associated with a CNV, to be routinely examined for the presence of an NDD, and for certain patients, stronger efforts need to be made to identify, treat, and manage the symptoms of these disorders (12, 31, 32).

The primary findings from this study also includes a significantly larger number of males than females identified in the CNV patient population. Additionally, CNV type was determined to be statistically non-significant in the ratio of male-to-female patients, though this may be attributable to variations in CNV sample sizes (CNV types with lower sample sizes having more extreme male-to-female ratios). Nonetheless, these findings conflict with those of other studies (33) who have identified a higher burden of large, rare CNVs in females than in males. The results also suggest that mortality is highest prenatally and within the first 2 years after pregnancy. In a clinical setting, this result may indicate a need to attend to patients more frequently in this period. Indeed this result also seems to attest to the positive outcomes of surgical interventions intended to correct life-threatening CNV disease phenotypes (34, 35).

Finally, we have identified that a large proportion of genes at four of the CNV loci with the highest coincidence of CHD and NDD is expressed in both the heart and the brain and that disruption of the genes will have negative consequences for various biological processes as identified by PANTHER. Disruption to these processes, as a consequence of disrupted gene expression in both the brain and the heart, likely impacts both CHD and NDD onset. We have also highlighted the potential impact of congenital heart disease on the developing brain as a secondary contributor to NDD onset.

### Strengths and Limitations

This study has a number of strengths which reinforce its conclusions. With a sample size of >700 patients from around Wales over a 20-year period, the data represents a wide cross-section of patients. As a result, trends in the data are less likely to be skewed by a narrow or niche sample. The extensive number of variables recorded in the gathering of data has also allowed for a wider analysis than might have otherwise been possible, and this has allowed us to take a more comprehensive approach to our analysis.

On the other hand, this investigation is limited in a number of ways. Principally, over 300 patients had to be excluded (Figure 1), and this reduced the total patient sample size by over one quarter. Furthermore, a significant limitation of this study centers on the decision to pursue analysis on only one or two CNVs per patient under the label of a primary CNV. This was done in the interest of maintaining a straightforward analysis and under the assumption that clinically relevant CNVs are more likely to contribute meaningfully to the variations in data than other lesser studied CNVs. Nonetheless, the trends identified are, in many cases, attributed to only one CNV and only account for the role of other CNVs where additional primary CNVs were identified in a patient. Furthermore, the small sample sizes for many of the CNVs identified can limit the reliability of the trends that can be identified within them. Our results, nonetheless, are in accordance with other published studies showing that cardiac anomalies are associated with atypical neurodevelopmental outcomes (13, 36–39).

One limitation of this study relates to the nature of its 20-year gathering period. As a result, the effect of CNVs on mortality in patients later in life cannot be examined with this data set. A previous study suggests that certain CNVs and CNV lengths globally are associated with higher mortality in patients at an extreme age range (over ~85 years) (40). Future research may involve monitoring the CNV patients throughout life in order to determine the effect that CNVs may play in long-term mortality. Additionally, while we highlight a hypothesis for the role that a CHD may play in contributing to NDD phenotype (Figure 5B), the relationship identified in this study is purely one of correlation, and further work is required to identify the precise mechanistic effect of congenital heart disease on neurodevelopment *in utero*. Finally, a meta-analysis of CNV patients from different cohorts is critical to establish the significant corelation between CHDs and NDDs in patients with CNVs.

Finally, while this study suggests that the CHD–NDD co-occurrence may be higher in CHD patients with a CNV, as compared to CHD patients with genetic origins not limited to copy number variations, the data gathered as part of this study has not been compared to that of other cohorts. Differences between cohorts and analysis procedure (e.g., age of patient at evaluation) have the potential to meaningfully confound the results of a comparative analysis, and such an analysis is beyond the scope of what this investigation aimed to address. Therefore, the data presented here, about an excess of CHD–NDD coincidences, should be viewed as preliminary findings. A meta-analysis of CNV patients from different cohorts, however, is critical to corroborate the significant correlation between CHDs and NDDs in patients with CNVs.

There is the potential that the geographically limited patient group will have biased the outcomes of this investigation. If it is the case that CHD and NDD occurrence is higher in Welsh populations in general, this may be reflected in their co-occurrence among Welsh CNV patients. However, at this time, there is no evidence to suggest that the CNV/CHD/NDD disease profiles of Welsh patients differ meaningfully from those of the wider UK or other populations.

### Public Health Implications

Despite the limitations of this study, it remains a novel first step in characterizing the relationship between CHD and NDDs in CNV patients, highlighting the need to address this relationship clinically. A future study should investigate at what time point, developmentally speaking, the presence of CNVs leads to atypical brain and heart development and identify other causal factors that can influence this process. Such research will be key in developing novel treatments for these diseases and in expanding our understanding of the association between genetics, heart function, and brain function during human development.

## Conclusion

This investigation, for the first time, establishes the burden of CNVs in children carrying CHDs across Wales and demonstrates a survival rate of 99%. Moreover, it constitutes a novel first step in characterizing the relationship between CHDs and NDDs in CNV patients, highlighting CNV-carrying patients with a CHD as a high-risk group for developing neurobehavioral abnormalities which can impact their quality of life. Hence, these children need a careful evaluation in terms of treatment and managing strategies. As this is a retrospective study, future experimental work should identify the underlying mechanistic pathways that lead to the atypical development of the brain and the heart and identify therapeutic targets. Additionally, future investigation is also required to cement the understanding of the role that CNV presence may play in increasing the likelihood of NDD onset in CHD patients.

## Data Availability Statement

The original contributions presented in the study are included in the article/Supplementary Material, further inquiries can be directed to the corresponding author/s.

## Ethics Statement

Ethical review and approval was not required for the study on human participants in accordance with the local legislation and institutional requirements. Written informed consent from the participants' legal guardian/next of kin was not required to participate in this study in accordance with the national legislation and the institutional requirements.

## Author Contributions

YAS conceived the idea, designed the study, takes full responsibility for the accuracy of the data analyses, and attests that all the authors listed meet the authorship criteria. LD, DT, SM, OU, and YAS were involved in the acquisition, analysis, or interpretation of data, critically revised and commented on the analyses and data interpretation, and revised the manuscript. DT takes responsibility for the integrity of the data. LD and YAS wrote and drafted the manuscript. All the authors critically revised the manuscript and approved the final version of the study.

## Conflict of Interest

The authors declare that the research was conducted in the absence of any commercial or financial relationships that could be construed as a potential conflict of interest.
