# Supplementary material for: Contribution of Congenital Heart Disorders Associated With Copy Number Variants in Mediating Risk for Brain Developmental Disorders: Evidence From 20-Year Retrospective Cohort Study
Source: Front Cardiovasc Med. 2021 Jul 15;8:655463. doi: 10.3389/fcvm.2021.655463 (PMC8319541; doi:10.3389/fcvm.2021.655463)
Supplement: Supplementary file 1 [file Data_Sheet_1.docx]

Supplementary Figure 1: Graph showing the distribution of patient referrals over 20years from Congenital Anomaly Register for Wales (CARIS).

Supplementary Figure 2: Scatter plot showing the co-relation between CHD-NDD co-incidence across CHD patients with CNV from referrals over 20 years in Congenital Anomaly Register for Wales (CARIS). Spearman correlation test output: 95% CI [0.4062, 0.8449], p < 0.0001, r = 0.6829.


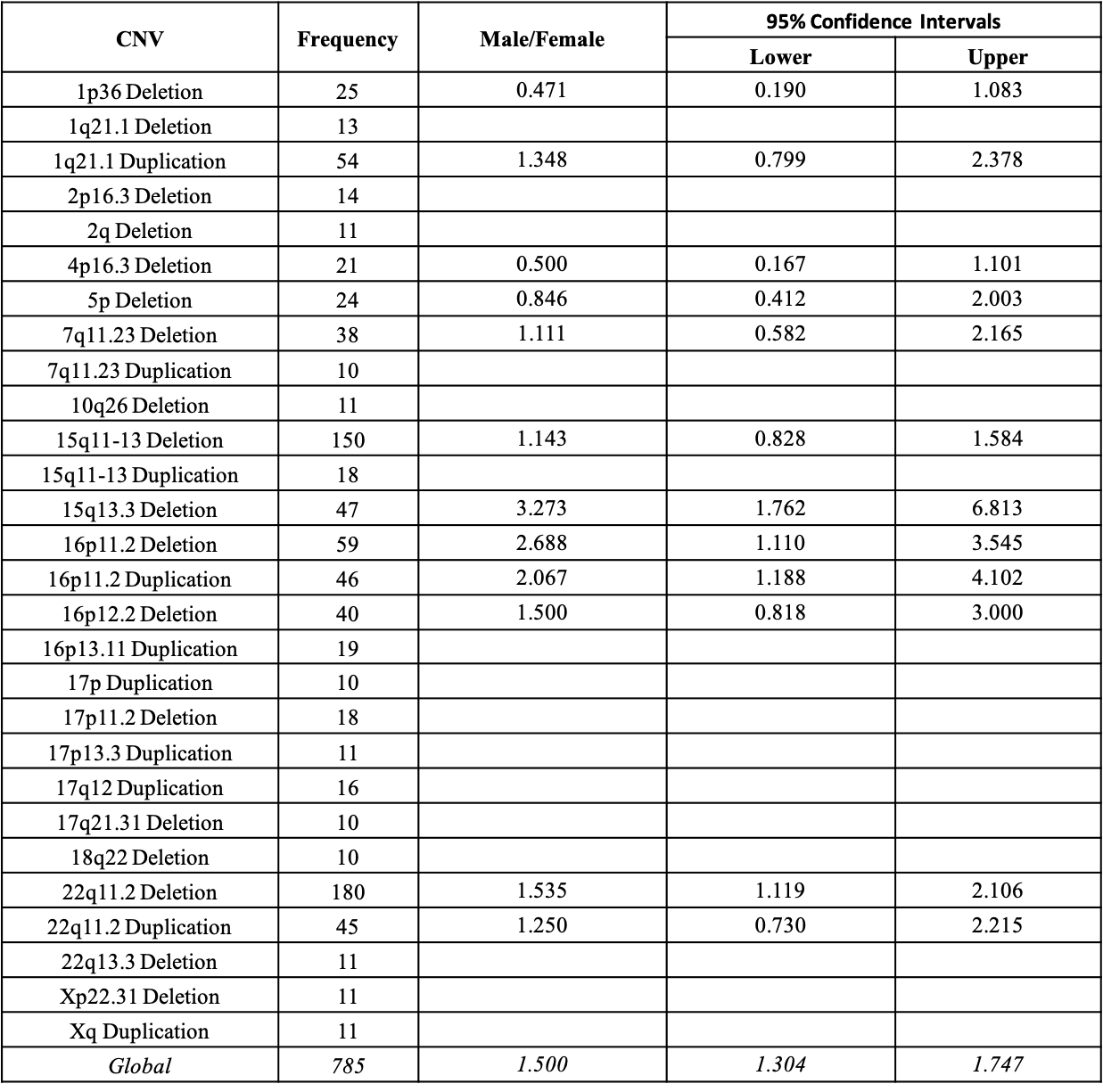


Supplementary Table 1: Table summarizing the 95% confidence intervals for CNV patient sex ratio, generated from bootstrapping with N = 1000 samples.


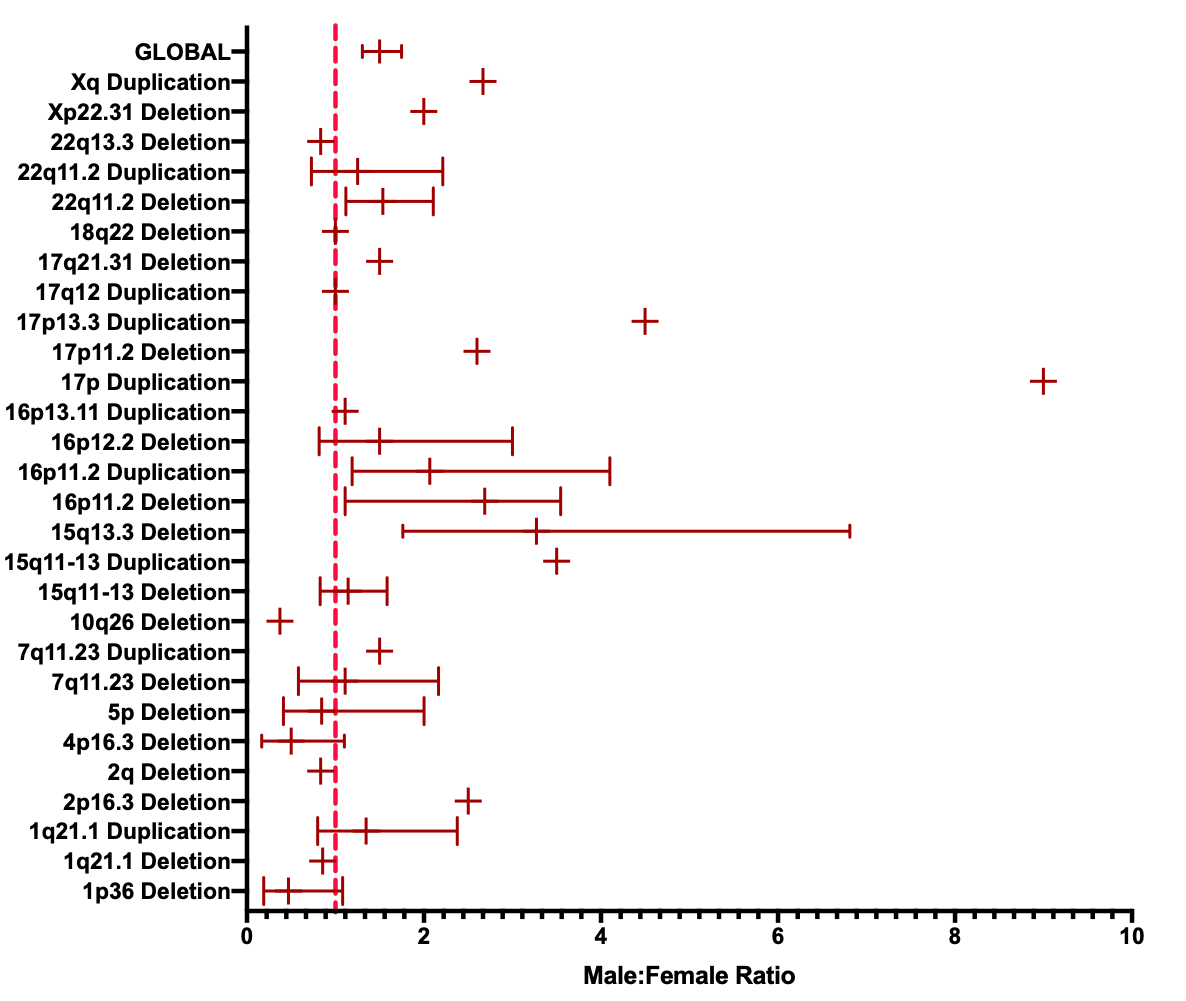


Supplementary Figure 3: Meta-analysis of cohort sex ratio on a linear axis, broken down by each CNV type, with 95% confidence intervals calculated for CNVs ≥20 patients (and Global value), via bootstrapping with N=1000 samples. CNV, copy number variant.

Supplementary Table 2: Tables of genes at top four chromosomal loci that mediate risk of NDD in patients with CHD carrying CNV, as used in PANTHER search.

Supplementary Table 3: Definitions of physiological functions identified by PANTHER search, as referenced in Fig. 5A, and source for definitions [all accessed June 2021].


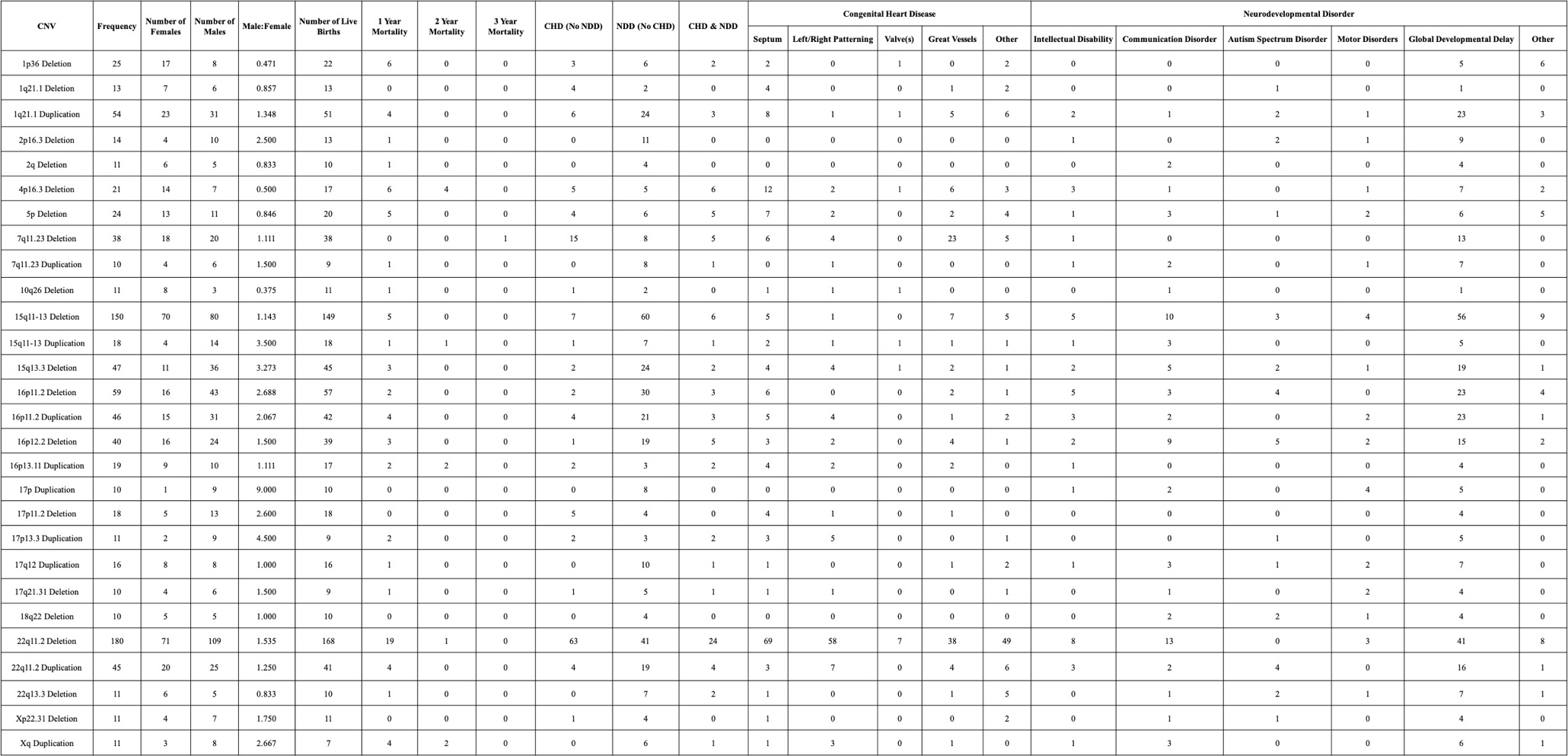


Supplementary Table 4: Table summarizing the variables associated with different CNVs as identified from Congenital Anomaly Register for Wales (CARIS).

**References:**

Battaglia, A., Carey, J.C., and South, S.T. (2015). Wolf-Hirschhorn syndrome: A review and update. *Am J Med Genet C Semin Med Genet* 169**,** 216-223.

Bergemann, A.D., Cole, F., and Hirschhorn, K. (2005). The etiology of Wolf-Hirschhorn syndrome. *Trends Genet* 21**,** 188-195.

Du, Q., De La Morena, M.T., and Van Oers, N.S.C. (2019). The Genetics and Epigenetics of 22q11.2 Deletion Syndrome. *Front Genet* 10**,** 1365.

Dutra, R.L., Pieri Pde, C., Teixeira, A.C., Honjo, R.S., Bertola, D.R., and Kim, C.A. (2011). Detection of deletions at 7q11.23 in Williams-Beuren syndrome by polymorphic markers. *Clinics (Sao Paulo)* 66**,** 959-964.

Mulle, J.G., Pulver, A.E., Mcgrath, J.A., Wolyniec, P.S., Dodd, A.F., Cutler, D.J., Sebat, J., Malhotra, D., Nestadt, G., Conrad, D.F., Hurles, M., Barnes, C.P., Ikeda, M., Iwata, N., Levinson, D.F., Gejman, P.V., Sanders, A.R., Duan, J., Mitchell, A.A., Peter, I., Sklar, P., O'dushlaine, C.T., Grozeva, D., O'donovan, M.C., Owen, M.J., Hultman, C.M., Kahler, A.K., Sullivan, P.F., Molecular Genetics of Schizophrenia, C., Kirov, G., and Warren, S.T. (2014). Reciprocal duplication of the Williams-Beuren syndrome deletion on chromosome 7q11.23 is associated with schizophrenia. *Biol Psychiatry* 75**,** 371-377.

Nguyen, J.M., Qualmann, K.J., Okashah, R., Reilly, A., Alexeyev, M.F., and Campbell, D.J. (2015). 5p deletions: Current knowledge and future directions. *Am J Med Genet C Semin Med Genet* 169**,** 224-238.
